# Changes of Morphology, Chemical Compositions, and the Biosynthesis Regulations of Cuticle in Response to Chilling Injury of Banana Fruit During Storage

**DOI:** 10.3389/fpls.2021.792384

**Published:** 2021-12-10

**Authors:** Hua Huang, Ling Wang, Diyang Qiu, Nan Zhang, Fangcheng Bi

**Affiliations:** ^1^Institute of Fruit Tree Research, Guangdong Academy of Agricultural Sciences, Key Laboratory of South Subtropical Fruit Biology and Genetic Resource Utilization, Ministry of Agriculture and Rural Affairs, Guangdong Provincial Key Laboratory of Tropical and Subtropical Fruit Tree Research, Guangzhou, China; ^2^Sericultural & Agri-Food Research Institute, Guangdong Academy of Agricultural Sciences, Key Laboratory of Functional Foods, Ministry of Agriculture and Rural Affairs, Guangdong Key Laboratory of Agricultural Products Processing, Guangzhou, China

**Keywords:** banana fruit, chilling injury, surface morphology, cuticle, biosynthesis regulations

## Abstract

The plant cuticle covers almost all the outermost surface of aerial plant organs, which play a primary function in limiting water loss and responding to the environmental interactions. Banana fruit is susceptible to thermal changes with chilling injury below 13°C and green ripening over 25°C. Herein, the changes of surface morphology, chemical compositions of cuticle, and the relative expression of cuticle biosynthesis genes in banana fruit under low-temperature storage were investigated. Banana fruit exhibited chilling injury rapidly with browned peel appearance stored at 4°C for 6 days. The surface altered apparently from the clear plateau with micro-crystals to smooth appearance. As compared to normal ones, the overall coverage of the main cuticle pattern of waxes and cutin monomers increased about 22% and 35%, respectively, in browned banana stored under low temperature at 6 days. Fatty acids (C_16_–C_18_) and ω-OH, mid-chain-epoxy fatty acids (C_18_) dominated cutin monomers. The monomers of fatty acids, the low abundant ω, mid-chain-diOH fatty acids, and 2-hydroxy fatty acids increased remarkably under low temperature. The cuticular waxes were dominated by fatty acids (> C_19_), *n*-alkanes, and triterpenoids; and the fatty acids and aldehydes were shifted to increase accompanied by the chilling injury. Furthermore, RNA-seq highlighted 111 cuticle-related genes involved in fatty acid elongation, biosynthesis of very-long-chain (VLC) aliphatics, triterpenoids, and cutin monomers, and lipid-transfer proteins were significantly differentially regulated by low temperature in banana. Results obtained indicate that the cuticle covering on the fruit surface was also involved to respond to the chilling injury of banana fruit after harvest. These findings provide useful insights to link the cuticle on the basis of morphology, chemical composition changes, and their biosynthesis regulations in response to the thermal stress of fruit during storage.

## Introduction

Banana is one of the most populated horticultural crops planted widely in the tropical and subtropical regions including south China. Banana contains abundant bioactive phytochemicals such as carbohydrates, vitamins, minerals, etc., being one of the most consumed fruit worldwide (Sidhu and Zafar, 2018). Low-temperature storage is one of the most efficient methods to preserve the food and crop quality. However, banana fruit is sensitive to temperatures, which exhibits chilling injury below 13°C and softening without normal yellow-colored under high temperature over 25°C (Yang et al., 2009; Hashim et al., 2012). The thermal sensitivity severely affects the fruit quality and their market values after harvest. The chilling injury symptoms of banana fruit mainly exhibit as browning in the vascular tissues and appearance and abnormal flesh softening (Wang et al., 2012). The chilling injury with browning changes is widely reported to be regulated by the reactive oxygen species (ROS), antioxidant activity, ATP level, and ion concentration in banana fruit. The temperature below chilling conditions disturbs the cellular ROS homeostasis, inducing the oxidation of polyphenols, accelerating energy dissipation, and ion flux (Huang et al., 2016; Liu et al., 2019). In addition, the integrity of the plasma membrane, which is pivotal for quality maintenance, is altered or damaged under ROS, temperature, or other stress stimulations during storage (Huang et al., 2019).

The plant cuticle constituted by complex lipids covers almost all aerial plant organs. The plant cuticle consists of soluble lipids as wax mixtures embedded in a cutin matrix. Cutin is prominently composed of C_16_ and C_18_ fatty acid monomers polymerized into the framework that harbors the various waxes (Philippe et al., 2020). The cuticular waxes are very-long-chain (VLC) fatty acids and their derivatives, which are tightly packed and aligned to act as impermeable flake obstacles, together with cyclic triterpenoids and sterols, form less hydrophobic amorphous zones (Jetter et al., 2008). The outermost lipid layer of cuticle plays multiple functions for the interactions between plant cells and environment (Yeats and Rose, 2013). The biosynthesis of cutin and wax compositions is well stated and regulated by a series of key enzyme genes. These genes are mainly within VLC fatty acid elongation, *n*-alkane pathway, alcohol pathway, ester formation, biosynthesis of triterpenoids, and sterols for the wax mixtures (Lewandowska et al., 2020), and also forming mid-chain hydroxyl and epoxy groups and polymerization process (Fich et al., 2016).

The accumulation of cuticular compositions is involved in response to abiotic and biotic stresses. Both the cuticular waxes and cutin were reported to play barrier or signal roles in the interactions between plant surface and the microorganisms (Isaacson et al., 2009; Hansjakob et al., 2010). As the primary function of cuticle is to limit the uncontrolled water loss, the biosynthesis of VLC components in waxes has been found to be significantly accumulated to adapt to the drought or salt stresses in plants (Kosma et al., 2009; Dimopoulos et al., 2020). Moreover, the high temperature or water-deficit environment will induce the accumulation of VLC esters (≥ C_36_) and triterpenoids in desert plant leaves (Schuster et al., 2016; Bueno et al., 2019). These reported results indicate that the surface cuticular components may have an effect on the temperature stress for plant tissues.

Recently, studies have tried to explore the effect of cuticular lipids on postharvest fruit quality changes (Heredia et al., 2019). The deficit biosynthesis of cutin monomers affected the fruit to resistant the microbial infection in tomato fruit after harvest (Isaacson et al., 2009). The triterpenoid fraction in fruit waxes was detected to exhibit a significant correlation with the postharvest behavior in blueberries (Moggia et al., 2016). The accumulation and crystal structure of cuticular waxes in orange fruit were found to be modified during storage under both low and room temperatures (Ding et al., 2018). Furthermore, the peel gloss, which was reported to be largely related to the cuticle changes (Petit et al., 2014), decreased with the ripening of banana fruit (Ward and Nussinovitch, 1996). Therefore, the cuticular lipids and their morphological appearance may play an important role in the ripening of fruit after harvest. The horticultural crops are usually stored under low temperatures to delay the shelf life and maintain their quality. So far, very few studies reported the cuticular waxes in varieties of bananas (Brian and David, 1985; Sampangi-Ramaiah et al., 2016), and the cutin mixtures of bananas have not yet been reported. Therefore, the cuticular lipids, including the cutin monomers and waxes, are necessary to be analyzed in detail as an update. Additionally, banana is a typical thermal-sensitive fruit, and the response of surface cuticular lipids to the chilling injury is also awaiting to be comprehensively investigated.

Banana fruit are usually harvested at green mature stage and turns to be yellow softening over 3 weeks during storage (Huang et al., 2013) but suffers chilling injury with most of browning appearance at 6 days (Huang et al., 2016). There are almost no obvious changes for the green mature banana fruit after 6 days stored under room temperature (Huang et al., 2013). Therefore, in the present study, banana fruit stored at 6 days under 4 and 25°C, respectively, were selected as the comparative samples. The chilling index and color changes of banana peel, surface morphology, and the changes of cuticle compositions including waxes and cutin monomers and the relative expression levels of cuticle-related genes were carried out in detail. The results obtained will broad insights on exploring the chilling injury of banana fruit on basis of the morphology, chemical, and molecular regulations of cuticle.

## Materials and Methods

### Plant Materials and Chemical Reagents

Banana fruits (*Musa* spp., AAA group, cv. Brazil) at physiological mature stage with green were harvested from an orchard in Guangzhou, Guangdong Province, P.R. China (23°30′N, 113°30′E). Harvested fruits were packed with polypropylene plastic bags (film thickness: 32 μm) and immediately transported to the laboratory within 2 h. Fruits were selected for uniformity of shape, color, and size, and rinsed the surface gently. Then fruits were placed into two groups randomly and packed in polyethylene bags (200 mm × 50 mm, 0.03 mm thickness, and three fruit per bag) and stored at 4 and 25°C, respectively. Six bags in total of 18 fingers from each treatment were randomly selected at 0, 2, 4, and 6 days during storage, and the chilling index and hue angle were evaluated. To isolate cuticular membranes, chemical reagents of analytical grade were prepared as previously described (Huang and Jiang, 2019). The peel tissues were sliced, frozen in liquid N_2_, and stored at − 80°C for further analysis.

### Determination of Chilling Injury Index and Hue Angle

The symptom of chilling injury was estimated by the extent of browning surface with different scales: “1” smooth surface without browning point; “2” slight browning with 1/4 of the fruit surface; “3” 1/4–1/2 browning area; “4” 1/2–3/4 browning area; and “5” > 3/4 browning area. The chilling injury index was calculated as: CI (chilling injury scale × corresponding fruit fingers within each class)/(number of total fruit × the highest scale) × 100.

The peel color degree was determined using a Minolta Chroma Meter CR-400 (Minolta Camera Co., Ltd., Osaka, Japan). Nine fruit fingers from each treatment at each stored stage and three points around the middle of each fruit were measured. Color was recorded as parameters of CIE L*, a*, and b* scales. In which, L indicates the lightness or darkness, a* indicates green to red, and b* donates blue to yellow. The overall color index was indicated by L* or general value of hue angle [*h*° = tan^–1^(b*/a*)].

### Scanning Electronic Microcopy

The surface characteristics of each organ of the two kinds of vegetables were observed using scanning electron microscopy (SEM). Briefly, small pieces (3 mm × 3 mm) of the peel tissues were picked and fixed in 2.5% glutaraldehyde in 0.2 M sodium phosphate buffer, pH 7.2–7.4 at 4°C overnight. The fixed samples were vacuum-infiltrated in the material on ice for 1 h. The samples were washed using sodium phosphate buffer and then dehydrated in a graded alcohol series with 30, 50, 75, 90, and 100% three times. After the samples were dried completely, the small pieces were mounted on aluminum stubs using a conductive double-sided adhesive tape prior to observation. The dried samples were coated with gold:palladium (60:40) at 30 mA using a LEICA EM ACE600 (Leica Microsystems, Wetziar, Germany) sputter coater, depositing an alloy of approximately 10 nm thickness. The characteristics of the sample surfaces were examined under a field emission SEM (JEOL JSM-6360LV, Tokyo, Japan) at 15 kV accelerating voltage.

### Isolation of Cuticular Membranes

The cuticles of banana fruit were isolated enzymatically following the protocol described by Huang and Jiang (2019). Fruit samples from each group at each stage were randomly selected. Peel discs were obtained by a puncher 1.2 cm in diameter near the middle position of the fruit. The punched discs were soaked in 10 mM citric acid buffer containing 1% (w/v) pectinase and 1% (w/v) cellulase (Beijing Solarbio Science & Technology Co., Ltd., Beijing, China). The cuticular membranes isolated from peel tissues were then washed by 10 mM sodium tetraborate decahydrate and distilled water in series. After that, cuticles were air-dried for further cuticular chemical analysis.

### Cuticular Wax and Cutin Monomer Extraction

The cuticular waxes and cutin monomers of banana fruit were extracted using the isolated cuticular membranes following the reported procedure previously (Huang, 2017). The dry and fine intact cuticular membranes isolated from banana fruit were immersed in chloroform completely. To better release the soluble waxes, the extraction was set with a moderate temperature at 40°C. The extraction for each sample was repeated three times, and each time for 2 min. After combining the extracted solution, *n*-tetracosane (Sigma-Aldrich, Shanghai, China) as an internal standard was added to help detect the accumulation of cuticular waxes. Gentle stream of nitrogen gas was used to dry the extracts for further analysis.

The above matrix membrane after removing waxes was subsequently depolymerized in boron trifluoride with methanol (BF_3_-methanol, 10%, ∼1.3 M) overnight at 70°C. Then, *n*-dotriacontane (Sigma-Aldrich, Shanghai, China) was added as an internal standard. Saturated aqueous sodium chloride solution and chloroform were added subsequently to extract the cutin monomers. The organic phase containing cutin monomers was collected and evaporated to dryness under a gentle stream of nitrogen gas for further analysis.

### Gas Chromatography-Mass Spectrometry

Prior to the analysis of the chemical composition of cuticular waxes and cutin monomers, the dry extracts prepared in the aforementioned manner were derivatized with pyridine (Shanghai Aladdin Bio-Chem Technology Co., Ltd., Shanghai, China) and N,O-*bis* (trimethylsilyl)trifluoroacetamide (BSTFA; Sigma-Aldrich, Shanghai, China) for 30 min at 70°C. To quantify wax and cutin monomer components, the extracts were analyzed using a capillary gas chromatography instrument equipped with a flame ionization detector (7820A, GC System; Agilent Technologies, Santa Clara, CA, United States), and on-column injection with a capillary column (30 m × 0.32 mm, DB-1 ms, 0.1 μm film; J&W Scientific, Agilent Technologies, Santa Clara, CA, United States). To separate cuticular wax compounds, 10 μL samples were injected at 50°C; after 2 min at 50°C, the temperature was raised to 200°C at 40°C min^–1^, held for 2 min at 200°C, and then raised to 320°C at 3°C min^–1^ and held for 30 min at 320°C. For separation of the cutin monomers, 10 μl samples were injected at 50°C; following 1 min at 50°C, the temperature was raised to 150°C at 10°C min^–1^, held for 2 min at 150°C, and then raised to 320°C at 3°C min^–1^ and held for 30 min at 320°C. The area under the peaks was compared with that of the internal standards to obtain the quantity of cuticular wax and cutin monomer components.

The chemical components were analyzed using a temperature-controlled capillary gas chromatography instrument equipped with a mass spectrometric detector (*m*/*z* 50–750, MSD 5975; Agilent Technologies, Santa Clara, CA, United States). Single compounds were identified based on their electron ionization mass spectra using authentic standards, the Wiley 10^th^/NIST 2014 mass spectral library (W10N14; John Wiley & Sons), or by interpretation of the spectra according to the retention times and/or by comparison with data from the literature (Holloway, 1982; Jetter et al., 2008) or online database (LipidWeb)^1^. The coverage was quantified against the amount of the internal standard, and the average chain length of the VLC acyclic compounds was calculated following the method reported by Huang (2017).

### RNA-seq Analysis and Reverse Transcription Analysis

Three biological replicates were performed for each sample. Total RNA was extracted and removed the contaminations with DNase I. Then, the purity and concentration of the RNA were checked by multifunction microwell plate detector (Shanghai Dobio Biology Technology Inc., Spark^®^, Tecan, Shanghai, China) and Agilent 2100 Bioanalyzer (Agilent Technologies, Santa Clara, CA, United States). Then the cDNA was synthesized by mixing with DNA polymerase I and RNase H. The samples were amplified and sequenced on Illumina HiSeq™ 4000 by Gene *Denovo* Co. (Guangzhou, China). The fine data were mapped to the reference genome using published banana data. The gene expression abundance was represented by an RPKM value. Differentially expressed genes (DEGs) were identified by the DESeq2^2^, which indicated the change of false discovery rate genes over twofold with statistical significance (false discovery rate, FDR < 0.05). DEGs in the pointed pathways were analyzed in detail.

The total RNA of banana peel tissues using a HiPure Plant RNA Mini Kit (Magen, Guangzhou, China) and cleaned with DNase I (TaKaRa, Otsu, Japan). The DNA-free RNA was used as the template for reverse-transcription PCR (RT-PCR). The first-strand cDNA template of each gene was synthesized using PrimeScript^®^ RT Master Mix (Perfect Real Time) template kit (DRR036A; TaKaRa, Otsu, Japan), and their concentrations were determined using a multifunction microwell plate detector (Shanghai Dobio Biology Technology Inc., Spark^®^, Tecan, Shanghai, China). Relative expression levels of the sequenced DEGs related to fatty acid elongation (*MaLACS*, *MaKCS*, *MaKCR*, and *MaECR*) and wax biosynthesis (*MaCER1* and *MaFAR*) were carried out by qRT-PCR. The reaction system was SYBR Premix Ex Taq Kit (DRR420A; TaKaRa, Otsu, Japan) where PCR conditions for the primers were 95°C for 30 s, 95°C for 5 s, and 60°C for 34 s, 40 cycles. The Primer Premier 6.0 software (Premier, Canada) was used to design the primers (Supplementary Table 1). The qRT-PCR was performed with SYBR^®^ Premix Ex Taq™ II (Takara, Otsu, Japan) in an ABI7500 Real-Time PCR System (Thermo Fisher Scientific, Waltham, MA, United States). Three independent biological replicates were used in the analysis.

### Statistical Analysis

Statistical analyses were performed using the IBM SPSS (version 23, IBM Corp., Armonk, NY, United States) and SigmaPlot 12.5 (Systat Software, Inc., San Jose, CA, United States). Comparison analyses were performed by one-way ANOVA, and significant differences were analyzed at a level of 0.05. SigmaPlot 12.5 was used to elaborate the graphs shown in the figures.

## Results

### Changes in Fruit Appearance and Morphology During Storage

**FIGURE 1:**
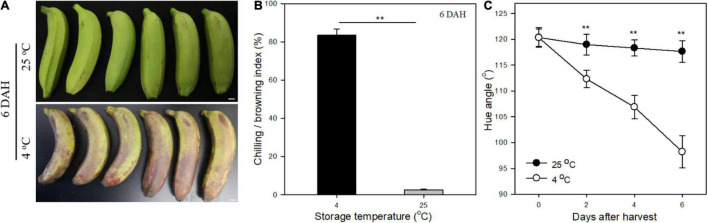
Changes of chilling injury of banana fruit under low temperature. **(A)** Appearance of fruit stored at 4 and 25°C for 6 days, and obvious browning occurred in banana at 4°C. **(B)** Chilling index indicated by browning area of fruit under low and room temperature at 6 days. **(C)** Changes in hue angle values following the storage time under different temperature storage. Data are presented as the mean ± SD. Significant differences detected by the *t*-test are indicated by *p < 0.05 and **p < 0.01, respectively.

Banana fruit exhibited an obvious browning appearance as the chilling injury symptom with an index over 80% of surface-browned at 6 days under 4°C after harvest (Figures 1A,B). The hue angle of fruit peel decreased rapidly from 120° to less than 100° following the chilling injury process, inducing the discoloration of green to brown at 4°C during storage (Figure 1C). In contrast, banana fruit stored at 25°C as control, the green peel appearance showed no obvious changes with trace of browning pot with most of them for mechanical damages during treatments after harvest (Figures 1A,B). The hue angle values of fruit exhibited relatively stable around 120° at 6 days stored at 25°C (Figure 1C). The green mature banana fruit changed slowly without ripening changes in the early storage period under room temperature, whereas suffered chilling injury rapidly as browning peel appearance under low-temperature storage (Huang et al., 2016).

**FIGURE 2:**
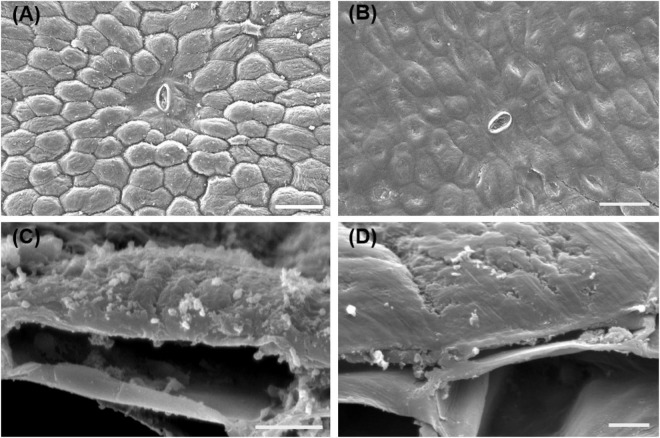
SEM observation of the surface changes of banana fruit during storage. **(A,C)** Surface microstructure changes sored at 25°C for 6 days. **(B,D)** Surface of banana fruit under 4°C at 6 days. Size bars: **(A,B)** 50 ţm and **(C,D)** 5 ţm.

The SEM observation showed the morphological changes of banana peel surface stored at 6 days under different temperatures. The epidermis of banana fruit consists of small square-, rectangular-, and gourd-shaped epidermal cells. These epidermal cells were outward forming numerous plateau ridges (Figure 2A). Similar to the previous anatomical analysis, the epidermal cells covered a thin layer of cell wall and cuticle on the outer surface of banana fruit (Amnuaysin et al., 2012). The outermost surface accumulated sparse small wax crystals (Figure 2C). After 6 days, the green mature banana fruit stored at 25°C showed clear cell shapes with crystals, indicating the well status for the morphological appearance in fruit (Figures 2A,C). However, the appearance of morphology was observed to be modified by the chilling injury under low temperature. Followed by the development of chilling-browning symptoms, the protruding epidermal cells collapsed and the surface of the fruit became flat (Figures 2B,D).

### Cuticular Chemicals Response to the Chilling Injury

The main pattern of cuticular components of cuticular waxes and cutin monomers was detected and quantitated thoroughly. The overall accumulation of cutin monomers was detected as 23.1 μg cm^–2^ in green mature fruit stored at 25°C, which was shifted to increase significantly (*p* < 0.05) as 31.2 μg cm^–2^ by chilling injury after 6 days (Table 1). Similarly, the total wax coverage was stimulated to increase from 30.0 to 36.6 μg cm^–2^ at 6 days under 25 and 4°C, respectively (Table 1). As a result, the ratio of total wax over cutin monomers showed no significant changes, which were about 1.2–1.3 (Table 1).

**TABLE 1:** The overall chemical composition of cutin monomers and waxes.

	25°C 6 days	4°C 6 days	*p* < 0.05	Unit
Wax yield	30.00 ± 3.24	36.61 ± 0.44	*	μg cm^–2^
Cutin yield	23.11 ± 3.92	31.21 ± 2.15	*	μg cm^–2^
Wax/cutin	1.32 ± 0.25	1.18 ± 0.09		Ratio
Aliphatics	19.94 ± 3.54	26.81 ± 1.87	*	μg cm^–2^
Cyclics	7.76 ± 0.92	8.37 ± 1.27		μg cm^–2^
Aliphatics/cyclics	2.56 ± 0.34	3.28 ± 0.75	*	Ratio
C_16_/C_18_ cutin monomer	0.21 ± 0.04	0.35 ± 0.05	*	Ratio
ACL	24.88 ± 0.10	23.76 ± 0.82		Carbons

The accumulation of total wax coverage and cutin monomers, the very-long-chain aliphatics, and cyclics (μg cm^–2^); the ratio of total wax versus cutin monomers, aliphatics versus cyclics, cyclics versus cutin, and monomers of C_16_ versus C_18_ in cutin; and weight average chain length (ACL) of aliphatics in waxes are shown. Data are given as mean value with SD (n = 5). *Indicates the significant deference at level of 0.05.

### Changes of Cutin Monomers

**FIGURE 3:**
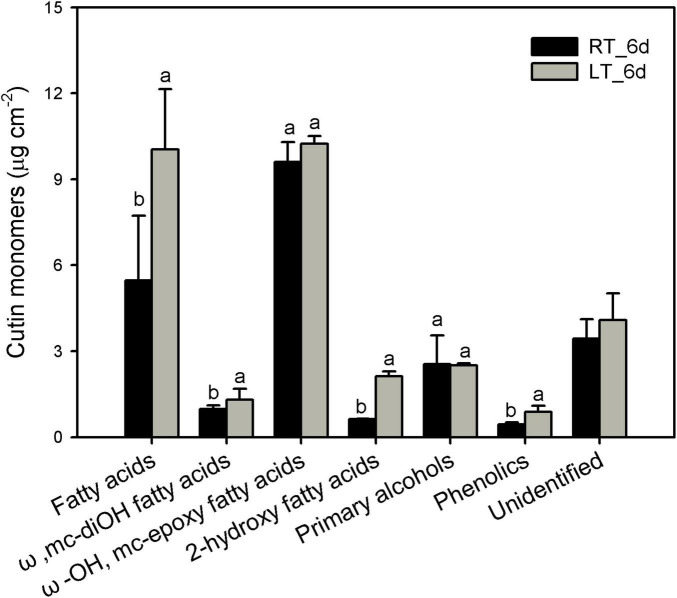
Chemical compositions of cutin monomers in banana fruit cuticle stored under 4°C (low temperature, LT) and 25°C (room temperature, RT) at 6 days. Data are given as mean ± SD (*n* = 5). Letters indicate the significant differences at the 0.05 level.

The cutin monomers in the series detected were prominently mid-chain epoxy ω-hydroxy fatty acids followed by fatty acids, primary alcohols, ω-mid-dihydroxy fatty acids, and 2-hydroxy fatty acids in banana fruit cuticles (Figure 3). The most abundant chain length of monomers was C_16_ and C_18_ fatty acids without or with branched hydroxyl or epoxy group. The predominant C_18_ monomer was 9,10-epoxy-18-hydroxyoctadecanoic acid, which showed no significant changes around 10 μg cm^–2^ in banana fruit cuticles under different temperatures (Figure 3 and Supplementary Table 2). Fatty acids without added groups accumulated in a wide range of chain-length distribution from C_16_ to C_30_, with most abundant of C_16_, C_18_, C_24_, and C_30_. The chilling stress stimulated the increase of C_16_- and C_18_-chain fatty acids remarkably, whereas C_24_ and C_30_ were relatively stable, resulting in the total fatty acids raised from 5.5 to 10.0 μg cm^–2^ (Figure 3 and Supplementary Table 1). The 9(10), 16-dihydroxyhexadecanoic acid was the main dihydroxy fatty acid increased slightly from 0.98 to 1.3 μg cm^–2^ (Figure 3 and Supplementary Table 2). Consequently, the prominent carbon chain of C_16_ over C_18_ in cutin monomers was shifted from 0.21 to 0.35 by chilling stimulation (Table 1). Noteworthy, another monomer of 2-hydroxy fatty acids accumulated with a chain length of C_22_–C_26_ with most abundant of C_24_, raised from 0.6 to 2.1 μg cm^–2^ after 6 days of low-temperature storage. Primary alcohols ranged from C_20_ to C_30_ with even-numbered carbon chains showed no obvious changes. In addition, cyclic monomers, predominantly coumaric acid and their derivatives, were also detected and increased slightly under low-temperature storage (Figure 3 and Supplementary Table 2).

### Changes of Cuticular Waxes

**FIGURE 4:**
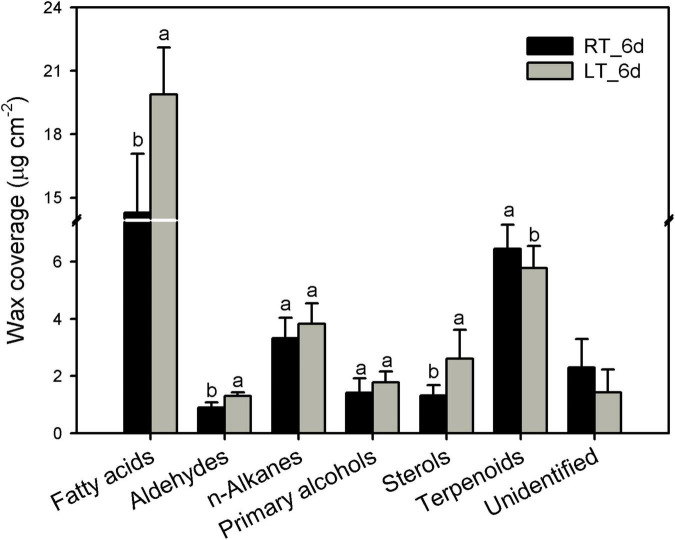
Chemical compositions of cuticular waxes in banana fruit cuticle stored under 4°C and 25°C at 6 days. Data are given as mean ± SD (*n* = 5). Letters indicate the significant differences at the 0.05 level.

The cuticular waxes in banana fruit cuticles were accumulated with a variety of typical VLC aliphatic and cyclic compounds. The VLC aliphatic pattern contained most abundant of fatty acids followed by *n*-alkanes, primary alcohols, and aldehydes. The cyclic pattern in waxes was dominated by pentacyclic triterpenoids and sterols (Figure 4 and Supplementary Table 3). Generally, the aliphatics accumulated higher than cyclics in banana fruit cuticles. The accumulation of aliphatics increased significantly to 26.8 μg cm^–2^ in banana fruit stored under 4°C compared with that of 19.9 μg cm^–2^ in fruit under room temperature (Table 1). The cyclics exhibited no obvious changes (about 8.0 μg cm^–2^) under both temperature storage. As a result, the ratio of aliphatics over cyclics was shifted from 2.6 to 3.3 (Table 1).

**FIGURE 5:**
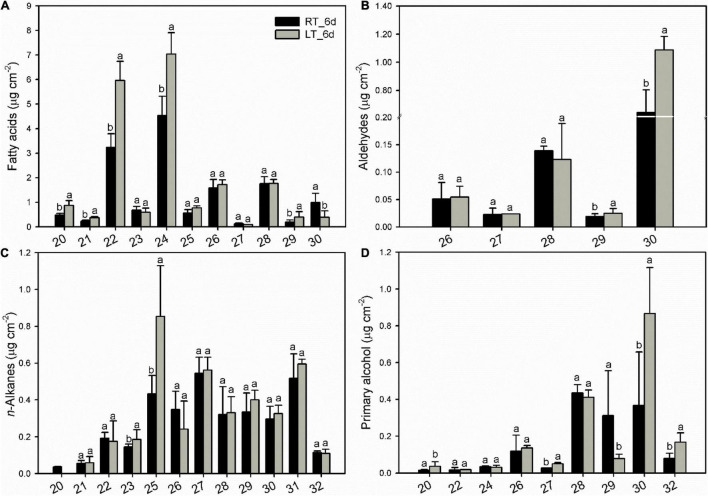
Changes in chain-length distribution and content of variety aliphatics in banana fruit cuticle stored under 4°C (low temperature, LT) and 25°C (room temperature, RT) at 6 days. **(A)** Fatty acids; **(B)** aldehydes; **(C)** *n*-alkanes; and **(D)** primary alcohols. Data are given as means ± SD (*n* = 5). Letters indicate the significant differences at the 0.05 level.

The various cuticular wax components accumulated into a homologous series. The VLC aliphatics (> C_18_) were detected within a fairly broad range of carbon chain lengths. Fatty acids as the most abundant aliphatic pattern ranged from C_20_ to C_30_ with most abundant of C_22_ and C_24_, which raised significantly under low-temperature storage at 6 days compared to that stored under 25°C (Figure 5A). Carbon chain ranged between C_20_ and C_32_ was detected for *n*-alkanes, almost no changes for all the *n*-alkane series of components, with an increase for C_25_ at 6 days under 4°C (Figure 5C). Aldehydes (C_26_ to C_30_) and primary alcohols (C_20_ to C_32_) accumulated with prominent of C_28_ and C_30_, which increased for C_30_ under low temperature (Figures 5B,D).

**FIGURE 6:**
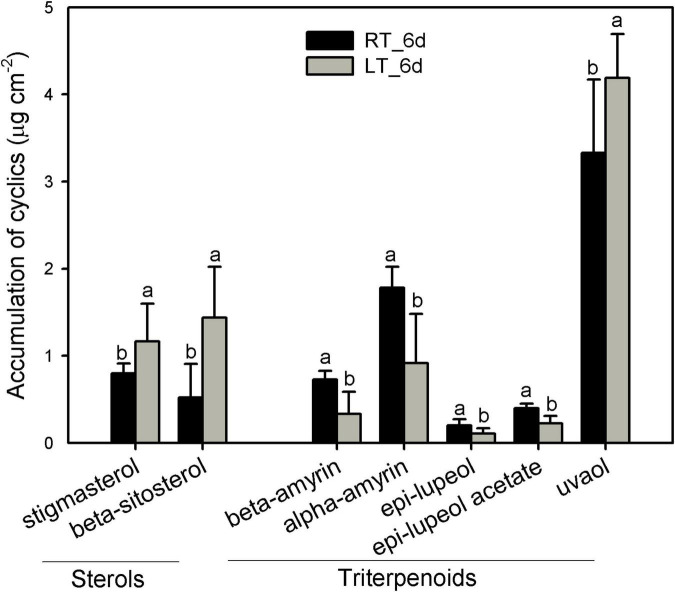
Changes in the components of triterpenoids in wax mixtures of banana fruit cuticle stored under 4°C (low temperature, LT) and 25°C (room temperature, RT) at 6 days. Data are given as mean ± SD (*n* = 5). Letters indicate the significant differences at the 0.05 level.

The pentacyclic triterpenoids dominated the cyclic pattern containing a variety of members with the most abundant being uvaol, followed by α-amyrin, β-amyrin, epi-lupeol, and epi-lupeol acetate. Sterols were comprised of β-sitosterol and stigmasterol (Figure 6 and Supplementary Table 3). After 6 days, besides uvaol, which showed a slight increase, the content of all the other triterpenoids was lower in fruit cuticles under 4°C than that under 25°C. In contrast, both β-sitosterol and stigmasterol were stimulated to increase under chilling temperature (Figure 6 and Supplementary Table 3).

### Response of the Biosynthesis of Cuticle

The gene response to the chilling injury on mRNA level was thoroughly analyzed using RNA-sequencing together with RT-qPCR. The results showed that in total of 16,707 DEGs were annotated with 9,420 DEGs (56%) downregulation and 7,287 DEGs (44%) upregulation in this study. The Gene Ontology (GO) annotation indicated that 7,576 DEGs (47.7% of total GO-annotated DEGs) were identified as “biological process” with 18 functional groups; 4,834 DEGs (30.5%) were annotated as “cellular component” with 14 functional groups; and 3,464 DEGs (21.8%) were involved in “molecular function” with 10 functional groups (Supplementary Figure 1). The Kyoto Encyclopedia of Genes and Genomes (KEGG) mapping analysis revealed that the DEGs were mainly involved in seven classes of various metabolism pathways (61.6% of total annotated DEGs), when compared to low temperature with room temperature storage (Supplementary Figure 2).

**FIGURE 7:**
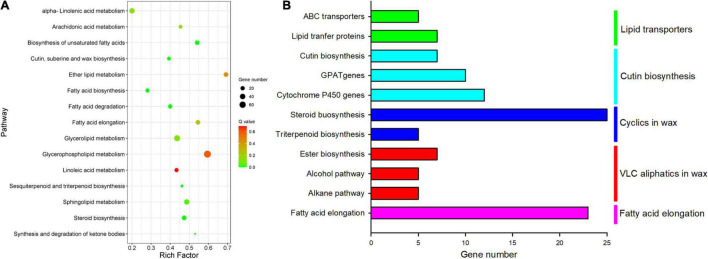
Enriched KEGG pathways involved lipids and cuticle metabolisms. **(A)** Scatter plot of enriched in lipid metabolisms; the rich factor is the ratio of the number of DEGs in each lipid pathway to the total DEGs involved in lipid metabolisms (0–1). **(B)** The number of differential cuticle-related genes in the five main cuticle biosynthesis pathways.

As cuticular constituents were mostly constituted by lipids, a total of 392 DEGs in 15 different pathways involved in lipid metabolism were detected. Fatty acid elongation, ether lipid metabolism, biosynthesis of unsaturated fatty acids, etc., were enriched markedly (rich factor > 0.5, Figure 7A). Further analysis revealed that 111 DEGs involved in biosynthesis and formation of cuticle were identified. These DEGs were mainly structural genes in five pathways for the biosynthesis of cuticle, i.e., fatty acid elongation, VLC alcohol and *n*-alkane pathways, cyclic waxes, cutin biosynthesis, and lipid-transfer proteins (Figure 7B).

**FIGURE 8:**
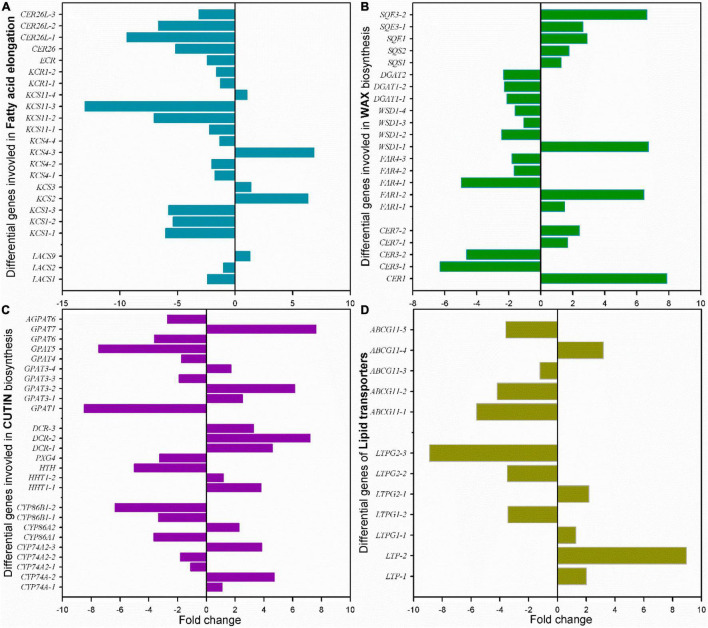
Changes of the main differential genes in cuticle biosynthesis of banana fruit during storage. **(A)** 23 DEGs in fatty acid elongation pathway, **(B)** 22 main DEGs in wax accumulation pathway, **(C)** 26 main DEGs in cutin monomer biosynthesis pathway, and **(D)** 12 DEGs for lipid-transfer proteins involved in cuticle forming were detected.

Among these cuticle-related genes, 23 DEGs were involved in fatty acid elongation including *MaLCAS1/2/9*, *MaKCS1/2/3/4/11*, *MaKCR1*, *MaECR*, and *MaCER26/26L* (Figure 8A and Supplementary Table 4). Besides numerous DEGs in sterol biosynthesis, 22 DEGs were identified as structure genes in wax biosynthesis respond to chilling injury, which were *MaCER1/3/7*, *MaFAR1/4*, *MaWSD1*, *MaDGAT1/2*, *MaSQS1/2*, and *Masqe1/3* (Figure 8B and Supplementary Table 4). About 26 DEGs in cutin monomer biosynthesis, *MaCYP74A*, *MaCYP74A2*, *MaCYP86A1/2*, *MaCYP86B1*, *MaHHT1*, *MaHHT*, *MaPXG4*, *MaDCR1/2/3*, *MaGPAT1/3/4/5/6/7*, and *MaAGPAT6* were differentially regulated (Figure 8C and Supplementary Table 5). And 12 DEGs of lipid transporters *MaLTP1/2*, *MaLTPG1/2*, and *MaABCG11*, which were potentially related to the transportation of cutin monomers and waxes were detected (Figure 8D and Supplementary Table 5).

## Discussion

The present study focused on characterizing the response of cuticle to the chilling injury in banana fruit under low temperature using room temperature as control. Banana is a typical horticultural crop that is sensitive to temperature changes during growing and postharvest storage. The chilling injury with browning of the peel tissues and discoloration, and informal firmness in banana fruit occurred after harvest. Low temperature disturbed the distribution of oxidation-related enzymes and the polyphenolics in plant cells, inducing the oxidation of polyphenolics in peel tissues (Huang et al., 2016). In addition, researchers proposed that the senescence of horticulture crops, including browning, discoloration, and softening, in banana fruit was also affected by the deficit of energy status. Under low temperature, the energy dissipation was accelerated following the chilling stimulation, thus inducing rapid browning and senescence in banana fruit (Liu et al., 2019). In addition to the subcellular changes, membrane integrity has been reported to be the earlier response to the ripening senescence and temperature injury for horticultural crops (Liang et al., 2020). The membrane lipids have been revealed as a pivotal pattern, especially the unsaturation degree of fatty acids, in adaptation to the temperature alterations (Zheng et al., 2011). The plasma membrane mainly constituted by mobile lipids with most of saturated and unsaturated C_16_ and C_18_ fatty acids. The chilling conditions could shift the ratio of saturated and unsaturated fatty acids in the plasma membrane and induce lipid peroxidation (Liu et al., 2020).

Similarly, besides the membrane lipids, the changes in cuticle, which are prominently lipid compounds, might also be involved in response to the temperature stress in banana fruit. The cuticle strength and stiffness showed largely to be affected by temperature changes (Domínguez et al., 2011). The high-temperature environment improved the accumulation of cyclic triterpenoids and VLC alkyl esters in desert plants (Schuster et al., 2016; Bueno et al., 2019). In addition, the cuticle structures, especially the arrangement of cuticular components, are dynamically modified by temperature changes, thus affecting the cuticle functions (Merk et al., 1997; Riederer, 2006). So far, few studies have reported the postharvest changes of cuticular compositions to affect the storage quality of fruit. The fruit ripening and postharvest cold storage were detected to accompany with alteration of cuticular waxes in blueberries (Moggia et al., 2016; Chu et al., 2018). However, the changes in cuticle of banana fruit in responding to low temperature are still unclear.

In banana fruit, the surface microstructure was apparently disturbed by low temperature after 6 days following the occurrence of chilling injury (Figure 2). The structural changes are usually accompanied by the shift of chemical compositions. For instance, the skin greasiness of apple fruit has been reported by the accumulation of liquid wax of propyl-, butyl-, pentyl-, and farnesyl-linoleate and oleate during storage (Yang et al., 2021). Similarly, low temperature induced changes in both cuticular waxes and cutin monomers in banana fruit cuticle followed by appearance changes. After 6 days of storage, low temperature largely downregulated the fatty acid elongation pathway in banana fruit (only five were upregulated out of 23 DEGs, Figure 8A and Supplementary Table 4). The fatty acid elongation enzymes (FAEs), such as long-chain acyl-CoA synthetases (LACSs), 3-ketoacyl-CoA synthases (KCSs), beta-ketoacyl-CoA reductases (KCRs), (3R)-3-hydroxyacyl-CoA dehydratases (HACDs), and enoyl-CoA reductase (ECR), are limit factors to regulate the elongation of VLC fatty acids (Samuels et al., 2008). In addition, CER26 or CER26L has also been identified to limit the biosynthesis of VLC fatty acids, particularly longer than C_30_ (Pascal et al., 2013). Compared with room temperature storage, most of the FAEs detected in DEGs were downregulated by chilling temperature. The downregulation of FAEs resulted in slowing down the accumulation of VLC fatty acids, particularly the chain length over C_28_. Accordingly, the accumulation of C_22_ and C_24_ as relatively short carbon chains in the VLC fatty acids in waxes increased in banana under low temperature (Figures 4, 5).

The cuticular waxes are stimulated to accumulate by the environmental stresses, i.e., the drought stress improved the accumulation of VLC *n*-alkanes with C_29_ and C_31_, or stimulated the increase of VLC alkyl esters in plant leaf or fruit cuticles (Kosma et al., 2009; Patwari et al., 2019; Dimopoulos et al., 2020). In banana fruit, an equal number of five genes were down- and upregulated, respectively, in the *n*-alkane and alcohol forming pathways. It was consistent with the relatively stable coverage of alcohols and *n*-alkanes, which only increased slightly for aldehydes with C_30_ and *n*-alkanes with C_25_ during storage (Figures 4, 5, 8B). One gene for each step in elongation and wax forming pathways of *MaLACS*, *MaKCS*, *MaKCR*, *MaECR*, *MaCER1*, and *MaFAR* was selected to further perform the qPCR analysis. The relative expression level of these genes was stimulated to increase by the low temperature in 4 days, but declined rapidly following the chilling injury (Supplementary Figure 3). In addition, changes in cyclic waxes, i.e., triterpenoids have been reported to be related to the postharvest quality of blueberries during storage (Moggia et al., 2016). In banana fruit, the sterols in cuticle of banana fruit increased obviously, whereas a decreasing trend was found for the triterpenoids under low temperature (Figure 6). Numerous EDGs were annotated as sterol biosynthesis, but only five candidates as *SQS* and *SQE* members were as the DEGs involved in biosynthesis of triterpenoids (Figures 6, 6B and Supplementary Table 4). In contrast to the accumulation of triterpenoids to reinforce the cuticle to adapt to the high-temperature condition in desert plants (Schuster et al., 2016), declined trend of triterpenoids was detected in banana fruit under low temperature. The decrease of triterpenoids might be one of the factors influencing the mechanical support of cuticle, thus inducing the collapsed surface for banana fruit during storage.

The cutin monomers polymerized as matrix is another main part of cuticle besides waxes. Genetic monitoring of the cutin deficiency induced the susceptible infection of microbial in tomato fruit (Isaacson et al., 2009). However, so far, postharvest changes of cutin monomers in fruit have been rarely reported. The cutin monomers of fatty acids without extra groups increased markedly, whereas the monomers with ω-OH mid-chain-OH or epoxy groups exhibited a declined trend under low-temperature storage in cherry fruit (Belge et al., 2014). On the contrary, an increasing trend for fatty acids without added groups or with 2-OH group was detected in banana fruit cuticle under low temperature (Figure 3). However, the monomers with ω-OH and mid-chain epoxy group, and abundant monomers as mono-functional fatty acids, exhibited no obvious changes during storage (Figure 3 and Supplementary Table 2). Both the carboxyl and hydroxyl groups in monomers are necessary to form polymers (Philippe et al., 2020). The cutin polymers in banana fruit might be rarely modified since the relatively stable monomers are added with functional groups. In addition, small molecules of phenolics as the aromatic pattern mainly including *p*-coumaric acid and their derivatives, which were common in plant cuticles, were also detected in banana fruit. These small molecules of phenolics are thought to be mainly embedded in the intracuticular layer together with cutin polymers (Fich et al., 2016). It might enhance the mechanical property for the cuticle, while this has been rarely cared and further work should be addressed. As shown in Figure 8C, besides the C_16_ and C_18_ fatty acids, the biosynthesis of cutin monomers regulated by *CYP74* and *CYP86* members, *HHT*, *DCR*, and *GPATs* exhibited homeostasis status with an equal number of 13 genes for downregulation and upregulation, respectively (Figure 8C and Supplementary Table 5). These results were highly consistent with the chemical results, which showed a relatively steady content of cutin monomers in banana cuticle during storage.

In addition, 12 lipid-transfer proteins involved in cutin monomer and wax secretion were also detected to be differentially regulated in banana cuticle under low temperatures (Figure 8D and Supplementary Table 5). Generally, two LTPs, five LTPG1/2, and five ABCG11 members were detected. LTPs have been widely found in flowering plants and are capable of exchanging lipids between membranes (Yeats and Rose, 2008). LTPGs are glycosylphosphatidylinositol-anchored lipid-transfer proteins, which have been identified as part of the export machinery in secreting cuticular waxes (DeBono et al., 2009; Kim et al., 2012). Under low temperatures, four LTPs and three LTPs were up- or downregulated, respectively, which might result in stable cuticular wax secretion. Accumulation of evidence indicated that ABCGs [ATP-binding cassette (ABC) subfamily G members] functioned to plant surface protecting against environmental stresses, and most of the reported members are involved in cutin formation (Elejalde-Palmett et al., 2021; Lee et al., 2021). In banana fruit, four of the five differential ABCG11 members were downregulated. The downregulation of ABCG members might slow down the secretion of cutin monomers; thus, the relatively stable cutin monomers, besides C_16_ and C_18_ fatty acids, were detected in banana fruit cuticle.

## Conclusion

**FIGURE 9:**
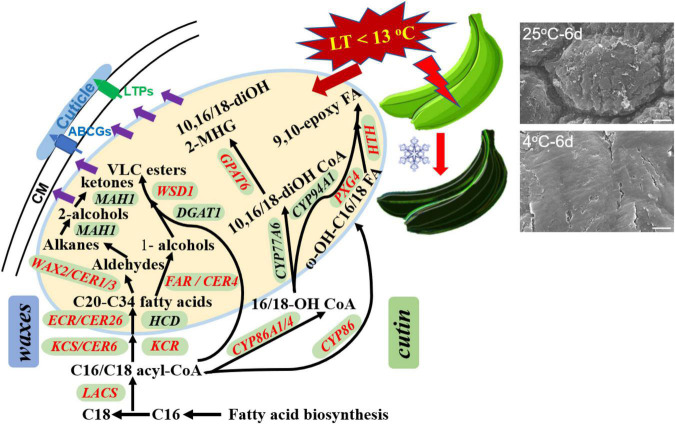
The overall changes in cuticle biosynthesis response to chilling temperature in banana fruit. The structural genes involved in cuticular wax and cutin monomer biosynthesis were differentially regulated (labeled in red) by low temperature in response to the appearance changes during storage.

This study in detail reported the changes of appearance morphology, response of chemical compositions, and their biosynthesis regulation genes in banana cuticle under low-temperature storage. Our study demonstrated that the appearance of banana fruit was altered apparently into flat paralleled with changes of cuticular wax and cutin monomers in cuticle stimulated by the chilling injury. Fatty acids of C_16_ and C_18_ as the monomers in cutin, and C_22_ and C_24_ fatty acids and cyclic sterols and triterpenoids in wax mixtures were the most changed cuticular components. To respond the chilling injury, a series of cuticle-related genes, i.e., *MaLACS*, *MaKCS*, *MaKCR*, *MaCER26*, *MaCER1/3*, and *MaFAR* in cuticular wax biosynthesis and *MaCYP86*, *MaCYP86A1/4*, *MaPXG4*, *MaHTH*, and *MaGPAT6* involved in cutin monomer accumulation, were differentially expressed in banana fruit (Figure 9). In addition, several lipid-transfer proteins, such as LTPG1/2 and ABCG11 members, were also differentially regulated. To our best knowledge so far, this study provides the initial exploration to link the cuticular response, on the basis of morphology, chemical, and biosynthesis regulation, to chilling stress on postharvest fruit. Further work on how the temperature affects the biosynthesis of cuticle and the potential functions for the response of cuticle to thermal changes and the molecular mechanisms will be comprehensively investigated.

## Data Availability Statement

The original contributions presented in the study are included in the article/Supplementary Material, further inquiries can be directed to the corresponding author/s.

## Author Contributions

HH designed and prepared the manuscript, performed most of the experiments, and prepared the draft of the manuscript. LW, DQ, and NZ contributed to part of the experiments and data analyses. FB took part in revising the manuscript and funding acquisition. All authors approved the final version of the manuscript.

## Conflict of Interest

The authors declare that the research was conducted in the absence of any commercial or financial relationships that could be construed as a potential conflict of interest.

## Publisher’s Note

All claims expressed in this article are solely those of the authors and do not necessarily represent those of their affiliated organizations, or those of the publisher, the editors and the reviewers. Any product that may be evaluated in this article, or claim that may be made by its manufacturer, is not guaranteed or endorsed by the publisher.

## Footnotes

1
https://www.lipidmaps.org/resources/lipidweb/lipidweb_html/index.html

2
http://www.bioconductor.org/packages/release/bioc/html/DESeq2.html
