# Supplementary material for: Changes of Morphology, Chemical Compositions, and the Biosynthesis Regulations of Cuticle in Response to Chilling Injury of Banana Fruit During Storage
Source: Front Plant Sci. 2021 Dec 10;12:792384. doi: 10.3389/fpls.2021.792384 (PMC8703112; doi:10.3389/fpls.2021.792384)
Supplement: Supplementary file 1 [file Data_Sheet_1.docx]

**Involvement of the changes of morphology, chemical compositions and the biosynthesis regulations in response to chilling injury of banana fruit under low temperature storage**

Hua Huang^1,*^, Ling Wang^2^, Diyang Qiu^1^, Nan Zhang^1^, Fangcheng Bi^1,*^

*^1^ Institute of Fruit Tree Research, Guangdong Academy of Agricultural Sciences; Key Laboratory of South Subtropical Fruit Biology and Genetic Resource Utilization*, *Ministry of Agriculture and Rural Affairs,Guangdong Provincial Key Laboratory of Tropical and Subtropical Fruit Tree Research, Guangzhou, 510640,  P. R. China*

*^2^ Sericultural & Agri-Food Research Institute Guangdong Academy of Agricultural Sciences, Key Laboratory of Functional Foods, Ministry of Agriculture and Rural Affairs, Guangdong Key Laboratory of Agricultural Products Processing, Guangzhou 510610, P. R. China*

*** Corresponding Author**

Email address: [huangw0109@gmail.com](mailto:huangw0109@gmail.com); bifangcheng@gdaas.cn

**Tabe S1**. Primers used for real-time PCR analysis

| Gene | F:5'-3’ | R:3'-5' |
| --- | --- | --- |
| *MaLACS* | GTCTGCCAAAGGGAGTAATGA | ATGTGAGCAAGTGGTAGGTATG |
| *MaKCS* | CGGTACAAGCTAAGGAAGGATG | TGCACCTGAAGGAGATTCTTG |
| *MaKCR* | TGCCATCAGCAGACACTTAC | CAGACACCATATGAGGGAATGG |
| *MaECR* | GTACTTGGGTCCTTTGGTTATCT | GCATATGTCTGGACTGGGTAAA |
| *MaCER1* | CATCCAAACACCCAGCTAAAC | GATGATGTCCCAGAGTGAAGAA |
| *MaFAR* | TGAACTCAACCGGAAGTACAAG | CATCCTCAACCTCTCCATGTTT |
| *MaActin* | TGGTATGGAAGCCGCTGGTA | CCTGCTGGAATGTGCTGAGG |

**Table S2**. Chemical composition of cutin monomers in detail of banana fruit cuticle at green mature fresh stage stored at room ambient about 25 ^o^C and low temperature at 4 ^o^C for 6 d, respectively. Data were given as mean values with SD (μg cm^-2^, n=5).

|  | |  | 25 ^o^C 6d | | | | | |  | 4 ^o^C 6 d | | | |
| --- | --- | --- | --- | --- | --- | --- | --- | --- | --- | --- | --- | --- | --- |
| **Fatty acids** |  | | |  |  | |  |  | | |  |  |  |
| **16** | 1.08 | | | ± | 0.45 | |  | 3.11 | | | ± | 1.24 |  |
| **18** | 0.37 | | | ± | 0.07 | |  | 2.16 | | | ± | 1.54 |  |
| **20** | 0.11 | | | ± | 0.03 | |  | 0.52 | | | ± | 0.52 |  |
| **22** | 0.78 | | | ± | 0.42 | |  | 0.59 | | | ± | 0.09 |  |
| **24** | 1.15 | | | ± | 0.53 | |  | 1.19 | | | ± | 0.13 |  |
| **26** | 0.38 | | | ± | 0.34 | |  | 0.71 | | | ± | 0.23 |  |
| **28** | 0.55 | | | ± | 0.25 | |  | 0.57 | | | ± | 0.07 |  |
| **30** | 1.06 | | | ± | 0.18 | |  | 1.19 | | | ± | 0.12 |  |
| **mid-OH-ω-Hydroxy fatty acids** | | | | | |  |  |  |  |  |  |  |  |
| **16** | 0.98 | | | ± | 0.13 | |  | 1.31 | | | ± | 0.38 |  |
| **mid-epoxy-ω-Hydroxy fatty acids** | | | | | | | | | | | | |  |
| **18** | 9.60 | | | ± | 0.70 | |  | 10.25 | | | ± | 0.27 |  |
| **2-Hydroxy fatty acids** |  | | |  |  | |  |  | | |  |  |  |
| **22** | 0.08 | | | ± | 0.07 | |  | 0.26 | | | ± | 0.10 |  |
| **23** | 0.11 | | | ± | 0.01 | |  | 0.33 | | | ± | 0.03 |  |
| **24** | 0.33 | | | ± | 0.10 | |  | 0.96 | | | ± | 0.07 |  |
| **25** | 0.05 | | | ± | 0.01 | |  | 0.32 | | | ± | 0.08 |  |
| **26** | 0.06 | | | ± | 0.02 | |  | 0.26 | | | ± | 0.05 |  |
| **Primary alcohols** |  | | |  |  | |  |  | | |  |  |  |
| **20** | 0.07 | | | ± | 0.02 | |  | 0.42 | | | ± | 0.59 |  |
| **22** | 0.90 | | | ± | 0.11 | |  | 0.70 | | | ± | 0.50 |  |
| **24** | 0.14 | | | ± | 0.07 | |  | 0.28 | | | ± | 0.26 |  |
| **26** | 0.24 | | | ± | 0.16 | |  | 0.25 | | | ± | 0.11 |  |
| **28** | 0.78 | | | ± | 0.43 | |  | 0.50 | | | ± | 0.38 |  |
| **30** | 0.43 | | | ± | 0.22 | |  | 0.30 | | | ± | 0.20 |  |
| **Phenolics** |  | | |  |  | |  |  | | |  |  |  |
| coumaric acid | 0.32 | | | ± | 0.07 | |  | 0.69 | | | ± | 0.21 |  |
| coumaric acid derivatives | 0.13 | | | ± | 0.02 | |  | 0.20 | | | ± | 0.07 |  |
|  |  | | |  |  | |  |  | | |  |  |  |
| **Unidentified** | 3.44 | | | ± | 0.69 | |  | 4.08 | | | ± | 0.93 |  |

**Table S3.** Chemical composition of waxes in detail of banana fruit cuticle at green mature fresh stage stored at room ambient about 25 ^o^C and low temperature at 4 ^o^C for 6 d, respectively. Data were given as mean values with SD (μg cm^-2^, n=5).

|  |  | 25 ^o^C 6d | | |  | 4 ^o^C 6 d | | |
| --- | --- | --- | --- | --- | --- | --- | --- | --- |
| **Primary alcohols** |  |  |  |  |  |  |  |  |
| 20 |  | 0.02 | ± | 0.00 |  | 0.04 | ± | 0.03 |
| 22 |  | 0.02 | ± | 0.01 |  | 0.02 | ± | 0.00 |
| 24 |  | 0.04 | ± | 0.00 |  | 0.03 | ± | 0.01 |
| 26 |  | 0.12 | ± | 0.09 |  | 0.14 | ± | 0.01 |
| 27 |  | 0.03 | ± |  |  | 0.05 | ± | 0.01 |
| 28 |  | 0.43 | ± | 0.05 |  | 0.41 | ± | 0.04 |
| 29 |  | 0.31 | ± | 0.24 |  | 0.08 | ± | 0.02 |
| 30 |  | 0.37 | ± | 0.29 |  | 0.87 | ± | 0.25 |
| 32 |  | 0.08 | ± | 0.03 |  | 0.17 | ± | 0.05 |
| **Aldehydes** |  |  |  |  |  |  |  |  |
| 26 |  | 0.05 | ± | 0.03 |  | 0.05 | ± | 0.02 |
| 27 |  | 0.02 | ± | 0.01 |  | 0.02 | ± | 0.00 |
| 28 |  | 0.14 | ± | 0.01 |  | 0.12 | ± | 0.07 |
| 29 |  | 0.02 | ± | 0.00 |  | 0.02 | ± | 0.01 |
| 30 |  | 0.64 | ± | 0.16 |  | 1.09 | ± | 0.10 |
| ***n*-Alkanes** |  |  |  |  |  |  |  |  |
| 20 |  | 0.04 | ± | 0.00 |  |  |  |  |
| 21 |  | 0.05 | ± | 0.02 |  | 0.06 | ± | 0.03 |
| 22 |  | 0.19 | ± | 0.03 |  | 0.17 | ± | 0.11 |
| 23 |  | 0.14 | ± | 0.02 |  | 0.19 | ± | 0.05 |
| 25 |  | 0.43 | ± | 0.10 |  | 0.85 | ± | 0.28 |
| 26 |  | 0.35 | ± | 0.10 |  | 0.24 | ± | 0.15 |
| 27 |  | 0.54 | ± | 0.09 |  | 0.56 | ± | 0.07 |
| 28 |  | 0.32 | ± | 0.15 |  | 0.33 | ± | 0.09 |
| 29 |  | 0.33 | ± | 0.10 |  | 0.40 | ± | 0.05 |
| 30 |  | 0.30 | ± | 0.07 |  | 0.32 | ± | 0.05 |
| 31 |  | 0.52 | ± | 0.14 |  | 0.60 | ± | 0.03 |
| 32 |  | 0.11 | ± | 0.01 |  | 0.11 | ± | 0.02 |
| **Fatty acids** |  |  |  |  |  |  |  |  |
| 20 |  | 0.48 | ± | 0.07 |  | 0.87 | ± | 0.19 |
| 21 |  | 0.23 | ± | 0.03 |  | 0.37 | ± | 0.06 |
| 22 |  | 3.23 | ± | 0.56 |  | 5.96 | ± | 0.78 |
| 23 |  | 0.67 | ± | 0.16 |  | 0.59 | ± | 0.16 |
| 24 |  | 4.54 | ± | 0.78 |  | 7.03 | ± | 0.87 |
| 25 |  | 0.55 | ± | 0.15 |  | 0.76 | ± | 0.09 |
| 26 |  | 1.58 | ± | 0.34 |  | 1.72 | ± | 0.20 |
| 27 |  | 0.13 | ± | 0.03 |  | 0.09 | ± | 0.00 |
| 28 |  | 1.76 | ± | 0.29 |  | 1.77 | ± | 0.16 |
| 29 |  | 0.19 | ± | 0.08 |  | 0.40 | ± | 0.22 |
| 30 |  | 0.99 | ± | 0.38 |  | 0.39 | ± | 0.26 |
| **Sterols** |  |  |  |  |  |  |  |  |
| stigmasterol |  | 0.80 | ± | 0.12 |  | 1.16 | ± | 0.43 |
| beta-sitosterol |  | 0.52 | ± | 0.39 |  | 1.44 | ± | 0.58 |
| **Triterpenoids** |  |  |  |  |  |  |  |  |
| beta-amyron |  | 0.73 | ± | 0.10 |  | 0.33 | ± | 0.26 |
| alpha-amyron |  | 1.78 | ± | 0.24 |  | 0.92 | ± | 0.56 |
| epi-lupeol |  | 0.20 | ± | 0.07 |  | 0.11 | ± | 0.06 |
| epi-lupeol acetate |  | 0.40 | ± | 0.05 |  | 0.22 | ± | 0.09 |
| uvaol |  | 3.33 | ± | 0.84 |  | 4.19 | ± | 0.50 |
|  |  |  |  |  |  |  |  |  |
| **Unidentified** |  | 2.29 | ± | 1.00 |  | 1.43 | ± | 0.80 |

**Table S4**. Differential genes at significant level involved in fatty acid elongation and wax biosynthesis in banana fruit. Results were carried out from the fruit stored at room temperature (RT) about 25 ^o^C versus that at low temperature (LT) at 4 ^o^C for 6 d, respectively. Data were given as mean values (n=3).

| **Gene ID** | **RT_fpkm** | **LT_fpkm** | **log2(FC)** | **Gene name** | **Pathway** |
| --- | --- | --- | --- | --- | --- |
| Ma08_g10580 | 4.78 | 0.91 | -2.40 | *LACS1* | Fatty acids to fatty acyl-CoA thioesters |
| Ma07_g28700 | 0.43 | 0.21 | -1.01 | *LACS2* |  |
| Ma02_g10050 | 2.56 | 6.23 | 1.28 | *LACS9* |  |
|  |  |  |  |  |  |
| Ma04_g22350 | 7.73 | 0.12 | -6.05 | *KCS1-1* | 3-ketoacyl-CoA synthase, involved in the first step biosynthesis of very-long-chain fatty acid (VLCFA) |
| Ma04_g32180 | 33.72 | 0.80 | -5.39 | *KCS1-2* |  |
| Ma05_g11040 | 22.33 | 0.41 | -5.78 | *KCS1-3* |  |
| Ma08_g04050 | 0.01 | 0.80 | 6.32 | *KCS2* |  |
| Ma01_g13760 | 0.44 | 1.13 | 1.38 | *KCS3* |  |
| Ma02_g10860 | 12.11 | 3.57 | -1.76 | *KCS4-1* |  |
| Ma04_g08880 | 2.14 | 0.52 | -2.04 | *KCS4-2* |  |
| Ma05_g08790 | 0.00 | 0.12 | 6.87 | *KCS4-3* |  |
| Ma09_g06710 | 27.50 | 10.90 | -1.34 | *KCS4-4* |  |
| Ma00_g00260 | 0.77 | 0.16 | -2.24 | *KCS11-1* |  |
| Ma04_g02980 | 11.75 | 0.09 | -7.03 | *KCS11-2* |  |
| Ma04_g24510 | 8.40 | 0.00 | -13.04 | *KCS11-3* |  |
| Ma07_g08400 | 80.64 | 166.98 | 1.05 | *KCS11-4* |  |
|  |  |  |  |  |  |
| Ma02_g07870 | 19.70 | 8.12 | -1.28 | *KCR1-1* | β-ketoacyl-CoA reductase |
| Ma03_g17840 | 3.43 | 1.13 | -1.61 | *KCR1-2* |  |
|  |  |  |  |  |  |
| Ma10_g26520 | 8.59 | 1.61 | -2.42 | *ECR* | enoyl-CoA reductase |
| Ma03_g26620 | 6.71 | 0.19 | -5.17 | *CER26* |  |
| Ma04_g21120 | 0.67 | 0.00 | -9.39 | *CER26L-1* |  |
| Ma05_g12350 | 13.66 | 0.14 | -6.64 | *CER26L-2* |  |
| Ma08_g06710 | 0.35 | 0.04 | -3.14 | *CER26L-3* |  |
|  |  |  |  |  |  |
| Ma11_g19740 | 0.00 | 0.23 | 7.87 | *CER1* | Alkane pathway |
| Ma01_g09200 | 26.30 | 0.33 | -6.30 | *CER3-1* |  |
| Ma09_g13090 | 11.60 | 0.47 | -4.64 | *CER3-2* |  |
| Ma06_g27260 | 3.08 | 9.89 | 1.68 | *CER7-1* |  |
| Ma08_g19040 | 4.04 | 21.57 | 2.42 | *CER7-2* |  |
|  |  |  |  |  |  |
| Ma09_g04360 | 3.84 | 10.87 | 1.50 | *FAR1-1* | Alcohol pathway |
| Ma09_g30420 | 0.03 | 2.31 | 6.44 | *FAR1-2* |  |
| Ma06_g20620 | 4.70 | 0.15 | -4.97 | *FAR4-1* |  |
| Ma09_g30400 | 2.81 | 0.88 | -1.67 | *FAR4-2* |  |
| Ma09_g30410 | 11.17 | 3.21 | -1.80 | *FAR4-3* |  |
|  |  |  |  |  |  |
| Ma03_g16670 | 0.01 | 1.41 | 6.72 | *WSD1-1* | Biosynthesis in alkyl esters |
| Ma04_g08220 | 0.13 | 0.02 | -2.44 | *WSD1-2* |  |
| Ma04_g08330 | 0.00 | 0.28 | -1.06 | *WSD1-3* |  |
| Ma04_g15300 | 54.91 | 18.06 | -1.60 | *WSD1-4* |  |
| Ma09_g28900 | 2.49 | 0.58 | -2.11 | *DGAT1-1* |  |
| Ma10_g21240 | 23.97 | 4.96 | -2.27 | *DGAT1-2* |  |
| Ma05_g26140 | 24.59 | 4.93 | -2.32 | *DGAT2* |  |
|  |  |  |  |  |  |
| Ma02_g14320 | 12.96 | 31.37 | 1.28 | *SQS1* | Triterpenoids biosynthesis |
| Ma04_g17820 | 1.61 | 5.49 | 1.77 | *SQS2* |  |
| Ma08_g04910 | 0.04 | 0.32 | 2.90 | *SQE1* |  |
| Ma08_g04870 | 0.56 | 3.47 | 2.62 | *SQE3-1* |  |
| Ma08_g04880 | 0.10 | 9.91 | 6.63 | *SQE3-2* |  |
| Ma05_g15840 | 2.68 | 0.33 | -3.04 | *CYP51-1* |  |
| Ma10_g12270 | 7.65 | 1.68 | -2.18 | *CYP51-2* |  |
| Ma10_g06670 | 5.22 | 0.29 | -4.15 | *CYP710A1* |  |

**Table S5.** Differential genes at significant level involved in cutin biosynthesis and lipid transporters in banana fruit. Results were carried out from the fruit stored at room temperature (RT) about 25 ^o^C versus that at low temperature (LT) at 4 ^o^C for 6 d, respectively. Data were given as mean values (n=3).

| **Gene ID** | **RT_fpkm** | **LT_fpkm** | **log2(FC)** | **Gene name** | **Pathway** |
| --- | --- | --- | --- | --- | --- |
| Ma05_g15840 | 2.68 | 0.33 | -3.04 | *CYP51-1* | Cytochrome 450 |
| Ma10_g12270 | 7.65 | 1.68 | -2.18 | *CYP51-2* |  |
| Ma10_g06670 | 5.22 | 0.29 | -4.15 | *CYP710A1* |  |
| Ma02_g15160 | 17.92 | 38.58 | 1.11 | *CYP74A-1* |  |
| Ma04_g38880 | 0.63 | 16.62 | 4.72 | *CYP74A-2* |  |
| Ma03_g26890 | 32.80 | 15.38 | -1.09 | *CYP74A2-1* |  |
| Ma05_g08520 | 61.23 | 17.46 | -1.81 | *CYP74A2-2* |  |
| Ma08_g04700 | 2.53 | 36.50 | 3.85 | *CYP74A2-3* |  |
| Ma06_g35740 | 0.17 | 0.01 | -3.64 | *CYP86A1* |  |
| Ma04_g32860 | 0.47 | 2.29 | 2.27 | *CYP86A2* |  |
| Ma04_g31950 | 0.23 | 0.02 | -3.32 | *CYP86B1-1* |  |
| Ma04_g35920 | 5.72 | 0.07 | -6.35 | *CYP86B1-2* |  |
|  |  |  |  |  |  |
| Ma04_g14490 | 0.01 | 0.19 | 3.81 | *HHT1-1* | diacid biosynthesis |
| Ma10_g17010 | 23.53 | 53.37 | 1.18 | *HHT1-2* |  |
| Ma03_g33500 | 0.54 | 0.02 | -5.01 | *HTH* |  |
| Ma09_g27850 | 182.83 | 19.19 | -3.25 | *PXG4* |  |
|  |  |  |  |  |  |
| Ma03_g14260 | 0.64 | 15.40 | 4.58 | *DCR-1* | involved in cutin polymerization |
| Ma10_g02420 | 0.47 | 69.89 | 7.22 | *DCR-2* |  |
| Ma10_g22410 | 0.06 | 0.55 | 3.28 | *DCR-3* |  |
|  |  |  |  | *1* |  |
| Ma08_g27730 | 11.97 | 0.03 | -8.49 | *GPAT1* | Synthesis of 2-monoacylglycerols |
| Ma11_g14660 | 0.04 | 0.25 | 2.51 | *GPAT3-1* |  |
| Ma03_g25580 | 0.16 | 11.11 | 6.15 | *GPAT3-2* |  |
| Ma07_g05670 | 0.61 | 0.16 | -1.89 | *GPAT3-3* |  |
| Ma08_g06170 | 0.72 | 2.36 | 1.72 | *GPAT3-4* |  |
| Ma01_g02100 | 1.04 | 0.31 | -1.73 | *GPAT4* |  |
| Ma02_g18170 | 24.31 | 0.14 | -7.47 | *GPAT5* |  |
| Ma03_g18650 | 1.70 | 0.14 | -3.60 | *GPAT6* |  |
| Ma10_g26580 | 0.00 | 0.20 | 7.62 | *GPAT7* |  |
| Ma11_g19270 | 12.27 | 1.88 | -2.70 | *AGPAT6* |  |
|  |  |  |  |  |  |
| Ma01_g10360 | 3.36 | 13.59 | 2.02 | *LTP602* | lipid transporters |
| Ma05_g00560 | 0.00 | 0.50 | 8.96 | *LTP* |  |
| Ma07_g24450 | 0.60 | 1.46 | 1.29 | *LTPG1-1* |  |
| Ma08_g00660 | 6.06 | 0.56 | -3.44 | *LTPG1-2* |  |
| Ma02_g14530 | 0.08 | 0.36 | 2.18 | *LTPG2-1* |  |
| Ma02_g19210 | 0.30 | 0.03 | -3.48 | *LTPG2-2* |  |
| Ma09_g26130 | 0.47 | 0.00 | -8.88 | *LTPG2-3* |  |
|  |  |  |  |  |  |
| Ma05_g10580 | 3.94 | 0.08 | -5.62 | *ABCG11-1* | ABC transporters for lipids |
| Ma05_g22190 | 10.96 | 0.60 | -4.18 | *ABCG11-2* |  |
| Ma06_g03830 | 0.52 | 0.22 | -1.22 | *ABCG11-3* |  |
| Ma06_g05720 | 0.02 | 0.21 | 3.19 | *ABCG11-4* |  |
| Ma10_g24650 | 0.20 | 0.02 | -3.58 | *ABCG11-5* |  |


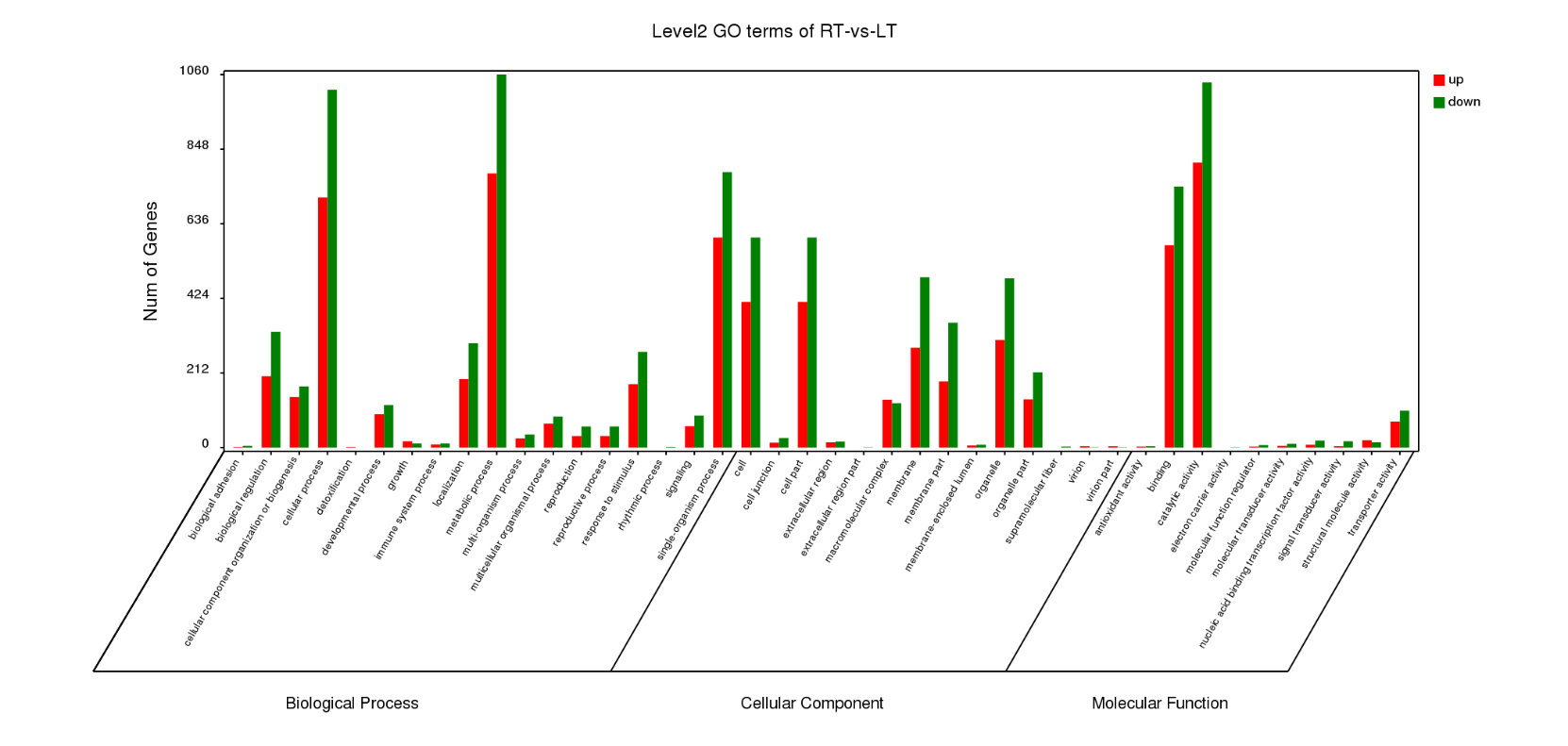


**Fig. S1** GO functional classification of the DEGs. The distributions are summarized in three main categories: biological process, cellular component and molecular function.

**Fig. S2** KEGG mapping analysis revealed various metabolism pathways as the most enriched responding processes.





**Fig. S3** Relative expression of selected genes involved in wax biosynthesis in banana fruit at different storage time after harvest.
